# Analysing the factor structure of the MAIA scale for pregnant women: Development of the MAIA-Preg

**DOI:** 10.1371/journal.pone.0322499

**Published:** 2025-05-07

**Authors:** Anna E. Crossland, Lydia B. Munns, Catherine E. J. Preston

**Affiliations:** University of York, United Kingdom; Hangzhou Normal University, CHINA

## Abstract

During pregnancy many elements of the bodily experience change, suggesting that measuring these constructs may require different instruments to those validated in the general population. This study reports an exploratory and confirmatory factor analysis exploration of the Multidimensional Assessment of Interoceptive Awareness (MAIA) in 716 pregnant women (mean gestation 26.4 weeks), from six different datasets who completed the MAIA online. Exploratory factor analysis condensed the questionnaire from a 32- to a 19- item scale, with five factor structure, which best fitted the data. Key subscales of Trust, Attention Regulation, Self-Regulation and Not Distracting remained robust. The one remaining item from the Noticing subscale loaded heavily with the Emotional Awareness subscale. Subscales of Body Listening, Not Worrying and Noticing did not load and therefore were excluded as factors. This led to the development of the scale referred to as the MAIA-Preg, which demonstrated a good fit with a confirmatory factor analysis along with good subscale reliability (ω= 0.73 -0.92), and measurement invariance for second and third trimesters. The MAIA-Preg was also found to be a good fit for separate non-pregnant (N = 396) and postnatal (N = 174) samples and thus provides a reliable and valid measure, providing nuanced information about the bodily experience in perinatal populations, allowing comparisons of changes to interoceptive sensibility the perinatal period.

## Introduction

The body experiences significant changes both externally and internally during pregnancy. Externally, the most obvious sign is an increase in the size of the abdomen where the baby is growing (baby bump), but other external changes are also very notable, for example to the appearance of the skin [1], increase in breast size [2] and swelling of the legs and feet [3]. There has been a substantial amount of research on the psychological impact [4–6] and measurement of external changes during pregnancy e.g., [7,8]. However, research [9] describes how a coherent experience of our own body is constructed from combining exteroception (sensory awareness from sensations external to the body; [10]) and interoception (the processes by which an individual senses, interprets, integrates, and regulates signals from within [them]self; [11]). During pregnancy a woman might perceive changes to sensations experienced by the general population, such as hunger and thirst [12,13] or pain [14,15], as well as direct physiological changes such as increased cardiac output [16] and reduced lung capacity [17].

Alongside changes to regular sensations, pregnant individuals might experience new sensations that are only notable during pregnancy, such as pelvic girdle pain [18,19], heavier, more sensitive and tingly breasts [20], and back pain due to stretching abdominal muscles and change in posture [21]. Women may also interpret bodily sensations differently during pregnancy, for example ongoing impactful nausea and sickness may be interpreted as less worrisome during pregnancy as it is expected and explained. In contrast, a sudden pain in the shoulder might instigate worry during pregnancy because it can be an indication of ectopic pregnancy, which wouldn’t be a concern for a non-pregnant population [22]. Pregnant women in focus groups described how they respond with acceptance to feelings like nausea, headaches and insomnia, whereas when recalling times before pregnancy they would feel frustrated, worried or upset at similar physiological experiences [23].

Our emotional state is intrinsically related to our physiological state as detected through interoception, for example when we feel strong emotions we may experience stronger and faster heartbeats. It is now understood that many mental health conditions are linked to differences in how we experience and interpret interoceptive sensations such that interoceptive sensibility is considered a transdiagnostic factor of mental ill-health [24,25], possibly due to exaggerated attention to or misinterpretation of internal bodily cues [26]. Vast physiological changes that occur during pregnancy, alongside an increased vulnerability to mental health issues at this time, highlight the importance of understanding how the interoceptive experience may change throughout pregnancy. Initial studies illustrate changes in interoceptive sensibility in pregnancy as well as links to mental well-being [27–32]. For example, those with poorer interoceptive experiences may be more likely to experience post-partum depression [27,32], and anxiety during pregnancy [28]. Not only this, but worry about interoceptive signals during pregnancy may moderate the negative relationship between body dissatisfaction during pregnancy and antenatal attachment, such that greater concerns about interoceptive signals reduces the association between body dissatisfaction and weaker antenatal bonds [30]. There has also been a potential direct relationship between interoception, specifically body trusting, and antenatal attachment [30,31], such that greater bodily (interoceptive) trust is associated with stronger bonds. This may also have an impact on a wide range of outcomes that are understood to relate to perinatal interoception, including antenatal attachment and breastfeeding [31], and perinatal mental health [31,32]. This is important because such outcomes have implications for infant development [33–35] as well as maternal well-being.

Measures of self-reported interoception allow for an understanding of how the individual feels subjectively about the recognition and perception of their bodily signals, irrespective of how objectively accurate they are. Self-reports also allow investigation of a wide range of bodily axes, rather than just heartbeats as is commonly used when measuring interoceptive accuracy (“the process of accurately detecting and tracking internal bodily sensations” P66; [36]). Therefore, self-reports could be considered a more useful test of interoception for identifying clinical status [37], allowing for a more accurate diagnosis and recognition of symptoms than measuring accuracy alone. The Multidimensional Assessment of Interoceptive Awareness (MAIA; [38]), is a multidimensional measure about how one interprets and trusts their positive and negative body feelings. It is one of the most commonly used scales to measure self-reported interoception, which is considered the traditional view of interoceptive sensibility [36], has been validated in many different languages [39] and is used for research relating to interoception in mental illness [e.g., 39] as well as during pregnancy e.g., [30].

The original MAIA scale is a 32-item self-report questionnaire measuring independent constructs of interoceptive sensibility, and is considered one of the most inclusive of the different validated measures, capturing the concept of interoceptive sensibility well within general populations [24]. Within the scale there are eight subscales, across independent dimensions of Noticing, Not Distracting, Not Worrying, Attention Regulation, Emotional Awareness, Self-Regulation, Body Listening and Trusting*.* The MAIA is multidimensional, and includes positive, negative and neutral bodily feelings from different domains as well as interpretation of bodily signals rather than simply recognising them. Evidence suggests it has good convergent and discriminant validity, and acceptable internal consistency and reliability in the general population [40]. However, the MAIA subscales of Not-distracting and Not-worrying were frequently reported with less than adequate internal consistency, which prompted the development of the MAIA-2 as an improved version with additional items [41]. Despite the development of the MAIA-2, the majority of research in pregnant samples continued to use the original MAIA [e.g., 26–30] with recent research finding that the Noticing subscale in addition to the Not-Worrying subscale, fell short of standardised cut-offs (Ω = 0.68 and Ω = 0.65, respectively) in this population [30]. Such poor internal reliability in a construct (Noticing) that was unchanged in the updated MAIA-2 due to consistent robust findings in the general population may suggest that the scale (either the MAIA or MAIA-2) is not robust for use in pregnant populations.

Although self-report measures may be well validated for measuring interoception in the general population, interoception may be more complex during pregnancy [26], and scales such as the MAIA may not capture the unique bodily changes that occur specifically during the perinatal period, or the differences in how those signals are interpreted. This is similar to how scales used to measure features of the exteroceptive experience do not access bodily changes that are specific to pregnancy [7,42]. For example, measures of body satisfaction validated in the general population are found to be answered differently in pregnant compared to non-pregnant samples [42] and do not capture the nuanced constructs related to pregnancy body change [7,42]. As interoceptive signals may be experienced and interpreted differently during pregnancy, many features of interoception specific to pregnancy do not feature in current measures such as the MAIA [38] or the updated MAIA-2 [41] as they are not intended to capture specific sensations during the prenatal period. Therefore, if the correct underlying constructs are not being captured by the scales being used in research, then the outcomes and assumptions based on them may be inaccurate. This may then have important implications for perinatal mental health through negatively impacting the replicability and validity of research.

Pregnancy interoception is a growing field of research that has predominantly measured self-reported interoception using the original MAIA scale [e.g., 26–30]. However, to date there is no statistically validated measure of interoception for pregnant populations, which limits interpretations of these findings. Therefore, this study aimed to examine the appropriateness of the MAIA as a measure of the latent variables underpinning interoceptive sensibility in a large sample of pregnant women. Because of the widespread use of the original MAIA in pregnancy research and that the adaptations in the MAIA-2 do not address issues apparent specifically for pregnant samples (as this was not its purpose) this study added to existing data (collected prior to MAIA-2 development [7]) to develop a pregnancy specific scale. This is important to allow for more accurate measurement of interoception during pregnancy and therefore a better understanding of the role interoception may play in other perinatal factors and postnatal outcomes, including those relating to mental health. To the best of our knowledge there is no other pregnancy specific measure of interoceptive sensibility.

## Method

### Participants

The MAIA was assessed in a total of four independent samples: two pregnant samples (sample 1 and 2), a postnatal sample (sample 3) and a non-pregnant sample (sample 4). Sample 1 was used for phase 1 (exploratory factor analysis, EFA), sample 2 was used for phase 2 (confirmatory factor analysis, CFA), with sample 3 and 4 used for phase 3 (validation in post-natal and non-pregnant samples). A total of 716 pregnant participants were recruited across 6 different projects, with ethical approval numbers 527, 174, 21121, 122, 2203 from the University of York [7,23,29–31], with data accessed January 2023-January 2024. All participants gave written consent, via electronic methods in Qualtrics. Authors did not have access to any identifying data during or after data collection. A median split following random number generation allowed the random allocation of pregnant women to sample 1 and 2. Pregnant and post-natal samples were recruited through online recruitment using social media, antenatal and postnatal groups, staff digest at the University of York and contacts from the research group. The ethnic makeup of those who reported ethnicity (N = 575) in the pregnant sample was 88% identifying as White, and 78% (N = 512) reported having some higher education (classed as some education from a university or a similar type of establishment). The non-pregnant group was recruited using social media and staff digests.

### Instruments

#### Multi-dimensional Assessment of Interoceptive Awareness (MAIA; [38]).

The MAIA is a 32-item self-report questionnaire measuring independent constructs of interoceptive sensibility. The scale consists of eight summated subscales, as outlined in Table 1:

**Table 1:** Subscales of the MAIA.

Subscale	Description	Number of items
Noticing	How much an individual is aware of their bodily sensations such as breathing and heart rate	4
Not-distracting	The tendency not to ignore or distract oneself from sensations of pain or discomfort from the body	3
Not-worrying	The tendency not to experience emotional distress or worry with sensations of pain or discomfort from the body	3
Attention regulation	The ability to sustain and control attention to bodily sensations	7
Emotional awareness	The awareness of the connection between body signals and emotional states	5
Self-regulation	The ability to regulate psychological distress by attention to bodily sensations	4
Body listening	The tendency to actively listen to the body for insight	4
Trusting	The experience of one’s body as safe and trustworthy	3

Responses are made on a 6-point Likert scale, in which participants indicate how often each statement applies to them generally in daily life, with responses from 0 (*never*) to 5 (*always*). The score for each subscale is calculated by the mean of its individual items, with no global score.

### Procedure

Participants across all four samples were directed to an online questionnaire delivered via Qualtrics (Qualtrics, Provo, UT) where they completed the MAIA [38]. The questionnaire took participants approximately 15 minutes to complete, including gathering demographic data on age, parity and gestation where appropriate, and in some cases were part of a bigger battery of other psychometric tests.

### Analyses

Questions 5–9 of the MAIA were reversed scored for analysis, as is required for accurate interpretation of the original scale [38], so that higher scores reflect stronger interoceptive skills. An accuracy check was conducted, to ensure that no scores were higher than 5 or below 0, to confirm no errors within the data. Analyses were conducted with the R statistical software (Version 3.4.3; R Core Team, 2017) and its *psych*, *lavaan* and *GPArotation* packages. The R scripts and datasets used in the analysis can be found in an online repository at https://www.openicpsr.org/openicpsr/project/210202/version/V1/view

### Phase 1: Exploratory factor analysis (EFA)

Sample 1 of pregnant women was used for the EFA. Suitability tests were initially conducted: Bartlett’s test of sphericity [43] was used to ensure that the data were suitable for factoring, with a significant outcome required [44]. The Kaiser-Meyer-Olkin (KMO) statistic was used to ensure suitability for factor analysis, with a value of 0.80 being considered ideal and 0.60 being adequate [45].

Common factor analysis was selected to satisfy the aims of identifying a latent factor structure [46], using a simple factor structure to allow each item to load onto only one factor in the EFA. Maximum likelihood estimation with an oblique oblimin rotation is considered an appropriate and useful method of representing the data when correlations are expected between variables [47], as is the case with the original MAIA scale [38]. The number of factors was determined by consulting a scree plot [48], undertaking parallel analysis, which is a technique to determine whether factors would provide more information about the latent construct than individual questions alone [49], and using consideration of Eigenvalues of > 1 [50] and also > .7 as a less stringent measure which was considered less likely to under-extract factors [51]. To ensure the outcome was driven by data and not pragmatic ideas or subjectivity of relying solely on a scree plot [44] or the arbitrary cut off of eigenvalues alone [47], analysis was carried out on all factors that were suggested as possibilities by each of the factor extraction methods.

A minimum factor loading threshold of 0.3 is used by convention [50], which demonstrates the correlation between the item and the factor, to ensure at least some shared variance between variables [44]. To ensure that the threshold was not too lenient .4 was also tested, as factors above .4 are considered particularly stable [52]. To be considered a strong factor, each item should also not load heavily onto another factor, so all factors with cross loadings were removed, as were items that did not load onto any factor, and the process repeated to refine the structure, until a simple solution was found. Convention indicates that the key global indices of good fit should be considered as well as local fit indices to describe how well the model represents the data [53], therefore fit indices of Comparative Fit Index (CFI), Tucker-Lewis Index (TLI), Standardised Root Mean Residual (SRMR), mean square error of approximation (RMSEA) were used to assess fit in line with accepted cut offs (see below).

### Phase 2: Confirmatory factor analysis (CFA)

#### Phase 2a: CFA.

To verify the best fitting factor structure outcome from the EFA on a new sample, a CFA was undertaken using sample 2. Chi square was used to compare the hypothesised model developed from the EFA with the new dataset using the maximum likelihood method, and the model can be regarded as acceptable if the chi-square test (χ²) is non-significant. Lower statistics for the ratio of chi-square to degrees of freedom (χ²/df) indicate a better model fit [54], however due to having a large sample size this should be approached with caution [55]. Average Variance Extracted (AVE) was also considered, to test for the amount of variance accounted for by the model, and Omega (McDonald’s ω) values alongside Cronbach’s alpha (α) were used to establish internal reliability. Measures of good fit (CFI, TLI, CI) and absolute fit indices of SRMR and RMSEA assessed the overall theoretical model against the data gathered. [56] suggest that the SRMR should be < 0.08, a RMSEA value of < 0.06 indicates good fit and 0.07–0.08 shows adequate fit. The CFI and TLI, which measure the incremental fit should be approaching 0.95 to be considered a good fit. All fit indices were compared against the original eight-factor, 32 item MAIA.

#### Phase 2b: Measurement invariance.

We wished to establish whether the scale was appropriate at different stages of pregnancy given that evidence suggests there are differences in the experience and interpretation of bodily sensations across the three trimesters [27,29]. Therefore, measurement invariance was calculated, to examine whether the factor structure fits equally well for women within their first, second and third trimester. Firstly, we checked for configural invariance to determine whether the factor structure was equivalent across groups. Next, we examined metric and scalar invariance. To reach scalar invariance (indicating that loading and intercepts are similar across groups), a difference in CFI of less than 0.01 indicated alongside a difference in RMSEA of less than 0.015 or a difference in SRMR of less than 0.030 is required [57]. However, this is considered conservative with others suggesting that the difference of less than 0.01 in CFI is sufficient to indicate scalar invariance [58].

### Phase 3: Validation in post-natal and non-pregnant samples

To verify whether the 5-factor model would be valid for post-natal and non-pregnant groups, a CFA was conducted on 174 post-natal women and 396 non-pregnant women using the same procedures as for the pregnant women from phase 2.

## Results

The demographic data for the different samples are shown in Table 2.

**Table 2:** Demographic data for the EFA and CFA samples, and comparison samples.

		Sample 1 (EFA)	Sample 2 (CFA)	Sample 3 (Post-natal)	Sample 4 (Non-pregnant)
N		358	358	174	396*
Age M (SD)		31.8 (4.61)	31.5 (4.62)	32.62 (4.63)	34.43 (6.81)
Weeks pregnant M(SD)		26.6 (8.85)	26.3 (9.04)		
Expecting multiple birth	Single	73%	77%		
	Multiple	0.8%	0.2%		
	Did not say	26%	22%		
Parity	Primiparous	58%	54%		
	Multiparous	39%	43%		
	Did not say	12%	4%		

Note: *The initial non-pregnant sample consisted of 466, however 44 non-pregnant women were removed as they were aged 50 + , so the age range of the sample matched. A further 26 were removed for missing data, leaving a total of 396.

### Phase 1: Exploratory factor analysis

Overall, 358 participants were included in the initial sample, however the Mahalanobis test (DF = 32) to check for outliers suggested that 17 participants were outside of the cut off (<0.001). This created a final sample of 341 on which the remaining checks and main analyses were undertaken. This is considered a large enough sample size regardless of which measure is taken to assess adequacy of sample size [51].

Bartlett’s test for homogeneity of variances (112.48; p < 0.001), indicates that there is significant heterogeneity in variances across the groups but the Kaiser-Meyer-Olkin statistic (KMO = 0.9 [45] and Bartlett’s test of sphericity (χ2 _(df=496)_=4891.4, p < .001) indicate that the items were still factorable. Normality tests were conducted on a dummy dataset; however, the Kolmogorov-Smirnov test showed a significant deviation from normality (D = 0.07, p = 0.04). Visual inspection of the histogram suggested a roughly symmetrical distribution, though some deviation from normality was observed. The number of potential factors according to the scree plot, parallel analysis and Eigenvalues of > 1 and > .7 were seven, three, three and five respectively, so all factors between seven and three were analysed to discover the optimum number for goodness of fit. Checks for sense were made, for example the seven factor scale with thresholds of 0.4 had good indices on all key measures, notably the cumulative variance (0.59) and TLI (0.97) were strong. However there were three subscales each with only two questions in, which would not make for valid or viable subscales [59], and was therefore excluded as a potential factor structure.

Table 3 illustrates the key indices of model fit for each potential factor structure. Table 4 illustrates the questions from the original MAIA, with their relative loadings on the different factors. Items with loadings under 0.3 are highlighted for removal, as are items that appear to load to > 0.3 but on more than one factor, therefore demonstrating cross loading, which was a criteria for removal of the item.

**Table 3:** Key indices of model fit, for both 0.3 and 0.4 thresholds, for each number of factors.

Model	Number of rounds		SRMR		TLI		CI (RMSEA)		RMSEA		Cumulative variance		No of items	
	0.3	0.4	0.3	0.4	0.3	0.4	0.3	0.4	0.3	0.4	0.3	0.4	0.3	0.4
**3 factor**	2	2	0.05	0.05	0.85	0.85	0.08-0.1	0.09-1	0.09	0.09	0.51	0.52	21	19
**4 factor**	2	3	0.03	0.03	0.93	0.96	0.05-0.07	0.04-0.06	0.06	0.05	0.53	0.58	19	16
**5 factor**	4	2	0.02	0.03	0.98	0.96	0.0-0.04	0.03-0.05	0.03	0.04	0.54	0.55	19	19
**6 factor**	3	2	0.03	0.03	0.91	0.92	0.05-0.06	0.04-0.06	0.05	0.05	0.53	0.56	26	23
7 factor*	2	2	0.03	0.02	0.94	0.97	0.03-0.05	0.02-0.05	0.04	0.03	0.55	0.59	26	22

Note: SRMR, TLI, CI, RMSEA and Cumulative variance all show the maximum results for that index. Rounds refers to the iterations of item removal that took place to identify the best factor structure.

*seven factor model had three subscales with ≤2 items

**Table 4:** Original MAIA question numbers, subscales and questions, together with the relative loadings and new subscales. Highlighted in grey demonstrates the question not loading and therefore being removed.

MAIA question number	MAIA subscale	MAIA question (MAIA-Preg question number, and any reverse scoring*)	Loading 1	Loading 2	Loading 3	Loading 4	Loading 5	MAIA-Preg subscale
1	Noticing	When I am tense I notice where the tension is located in my body. (10)	-0.08	-0.01	0.17	0	**0.33**	**Emotional Awareness**
2		I notice when I am uncomfortable in my body.	0.29	-0.02	0.18	0.04	-0.17	
3		I notice where in my body I am comfortable.	0.19	-0.04	0.30	-0.10	-0.06	
4		I notice changes in my breathing, such as whether it slows down or speeds up.	0.26	-0.08	0.18	0.20	-0.01	
5	Not distracting	I do not notice (I ignore) physical tension or discomfort until they become more severe. (1 *)	**0.44**	0.07	-0.12	0.14	0	**Not distracting**
6		I distract myself from sensations of discomfort. (2 *)	**1**	-0.01	0.02	-0.02	0	**Not distracting**
7		When I feel pain or discomfort, I try to power through it. (3 *)	**0.4**	-0.03	0.09	0.08	-0.04	**Not distracting**
8	Not worrying	When I feel physical pain, I become upset.	0.10	-0.01	0.14	0.26	-0.16	
9		I start to worry that something is wrong if I feel any discomfort.	0.00	-0.01	0.21	0.19	-0.24	
10		I can notice an unpleasant body sensation without worrying about it.	-0.23	0.20	0.18	-0.10	0.23	
11	Attention regulation	I can pay attention to my breath without being distracted by things happening around me. (4)	0.04	0.21	-0.01	**0.51**	-0.16	**Attention regulation**
12		I can maintain awareness of my inner bodily sensations even when there is a lot going on around me. (5)	0.03	-0.11	-0.01	**0.75**	0.07	**Attention regulation**
13		When I am in conversation with someone, I can pay attention to my posture. (6)	-0.02	-0.07	0.04	**0.66**	0.03	**Attention regulation**
14		I can return awareness to my body if I am distracted. (7)	0.05	0.08	0.02	**0.72**	0.08	**Attention regulation**
15		I can refocus my attention from thinking to sensing my body. (8)	-0.04	0.09	-0.02	**0.76**	-0.07	**Attention regulation**
16		I can maintain awareness of my whole body even when a part of me is in pain or discomfort. (9)	-0.05	0.02	0.18	**0.56**	0.07	**Attention regulation**
17		I am able to consciously focus on my body as a whole.	0.22	0.14	0.35	0.01	0.05	
18	Emotional awareness	I notice how my body changes when I am angry. (11)	0.07	0.07	-0.03	0	**0.6**	**Emotional Awareness**
19		When something is wrong in my life I can feel it in my body. (12)	-0.06	-0.02	0.01	0.03	**0.7**	**Emotional Awareness**
20		I notice that my body feels different after a peaceful experience.	0.51	-0.07	0.03	-0.06	0.35	
21		I notice that my breathing becomes free and easy when I feel comfortable.	0.54	-0.09	0.10	-0.08	0.32	
22		I notice how my body changes when I feel happy/ joyful. (13)	0.06	0.22	-0.05	0.17	**0.39**	**Emotional Awareness**
23	Self-regulation	When I feel overwhelmed I can find a calm place inside.	0.24	0.26	0.20	0.01	0.22	
24		When I bring awareness to my body I feel a sense of calm. (14)	-0.04	**0.48**	0.14	0.09	0.19	**Self-regulation**
25		I can use my breath to reduce tension. (15)	0.01	**0.94**	0.01	-0.04	-0.01	**Self-regulation**
26		When I am caught up in thoughts, I can calm my mind by focusing on my body/breathing. (16)	0.02	**0.69**	0.04	0.15	0.06	**Self-regulation**
27	Body listening	I listen for information from my body about my emotional state	0.48	0.13	0.06	0.16	0.18	
28		When I am upset, I take time to explore how my body feels.	0.48	0.32	0.14	0.32	-0.03	
29		I listen to my body to inform me about what to do	0.41	0.48	0.06	0.04	-0.09	
30	Trusting	I am at home in my body. (17)	0.02	-0.03	**0.84**	0.08	-0.06	**Trust**
31		I feel my body is a safe place. (18)	0	-0.01	**0.95**	-0.02	-0.02	**Trust**
32		I trust my body sensations. (19)	0.02	0.15	**0.7**	-0.03	0.14	**Trust**

Note: Bold refers to the questions that loaded about 0.3 threshold on only one factor, and therefore remained. Factor loadings for only the questions remaining in MAIA-Preg can be found in S2.

**Fig 1:**
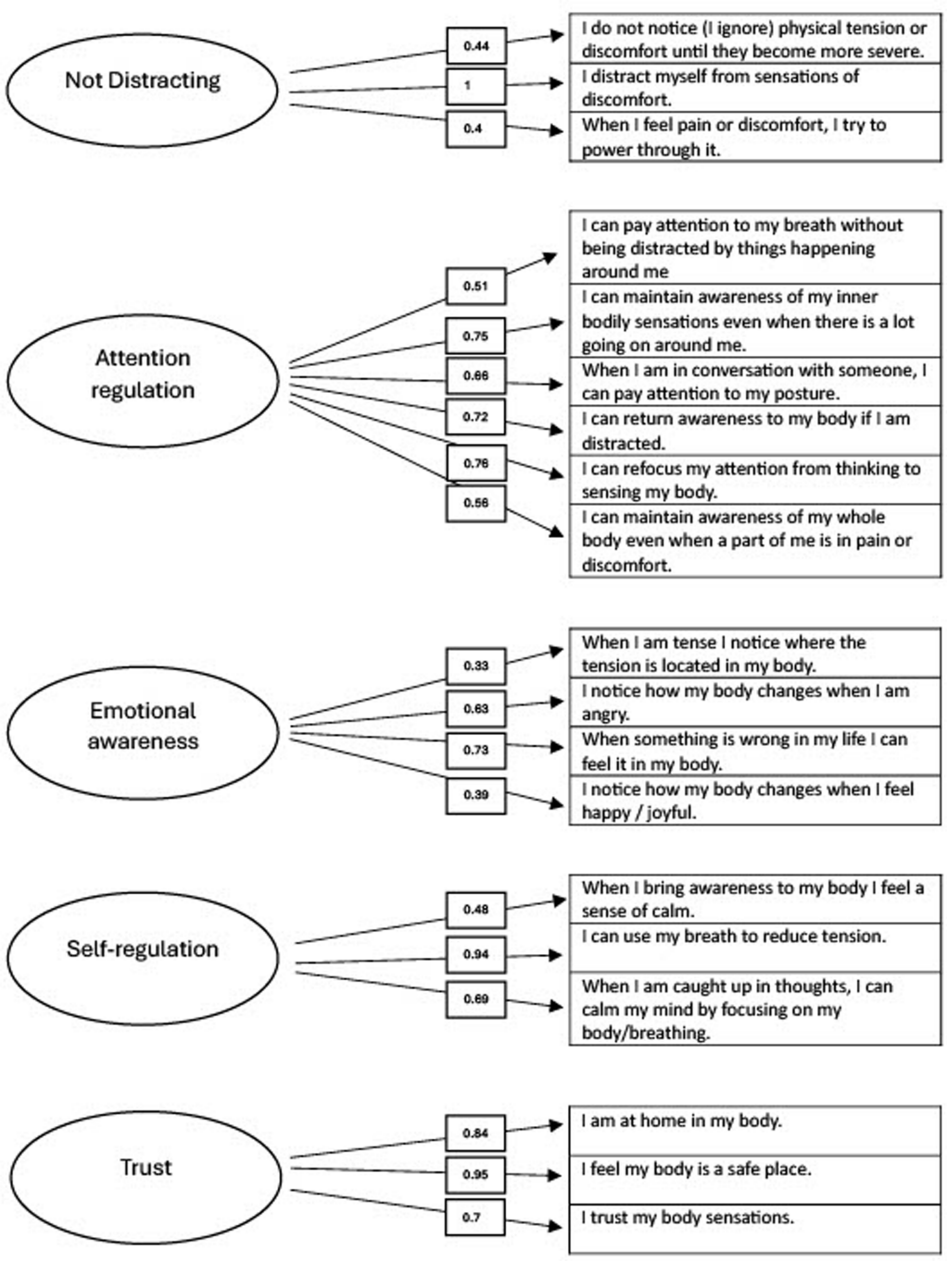
MAIA-Preg subscales and loadings.

From this analysis the researchers concluded that the five-factor model, with 19 items retained (now referred to as the MAIA-Preg, shown in Figs 1 and S1 File) was the best fitting factor structure compared to all other alternatives from the EFA as well as the original eight-factor 32-item model. This suggests that interoception manifests as a slightly different construct during pregnancy.

The MAIA-Preg accounted for 54% of the total variance, which is within an acceptable level [60], with each subscale separately accounting for 14% (Not Distracting, Emotional Awareness), 20% (Self-regulation), 23% (Trust) and 30% (Attention) of the variance. The five factor structure was strongest compared to all other feasible factor structures on all goodness of fit measures (see Table 3), with ten of the 19 questions loaded to > .7 with a further five questions loading to > .5. Most of the questions remain within the same subscales as the original scale, with the Trust, Attention Regulation, Self-Regulation and Not Distracting subscales proving robust, with Chronbach’s alpha (α) and McDonald’s Omega (ω) indicating high internal consistency (ω = 0.67–0.89). Analysis of internal reliability highlighted that subscales of Trusting and Attention Regulation have particularly high internal consistency, and Not Distracting and Emotional Awareness displaying low consistency. This potentially suggests that the items within the Not Distracting and Emotional Awareness subscales, although still relevant to pregnant women, are either not representing a homogeneous latent variable or that the items do not illustrate the construct strongly, shown in Table 5. However, the Not Worrying, Noticing and Body Listening subscales did not appear strongly for pregnant women, with none of the questions remaining in the Body Listening and Not Worrying subscales. Whilst one remaining question in the Noticing subscale (“When I am tense I notice where the tension is located in my body”) loaded together with the questions in the Emotional Awareness subscale, this item was also deemed to represent emotional awareness of sensations, reflecting an awareness of feelings of tension in the body. For this reason, the original factor name (Emotional Awareness) was retained.

**Table 5:** Internal reliability of the subscales of the MAIA-Preg.

	Alpha (Chronbach’s)		Omega (McDonald’s ω)	
	EFA	CFA	EFA	CFA
Not distracting	0.63	0.69	0.67	0.73
Attention regulation	0.85	0.88	0.88	0.92
Emotional Awareness	0.64	0.69	0.67	0.75
Self-regulation	0.84	0.82	0.85	0.83
Trusting	0.89	0.89	0.89	0.90

Subsequently a CFA was conducted to verify the structure of the MAIA-Preg with a new sample, and to compare the five factor MAIA-Preg structure with the original eight factor MAIA.

### Phase 2a: Confirmatory factor analysis

For the CFA, 358 participants were included in the initial sample, however the Mahalanobis test to check for outliers suggested that 24 participants were outside of the cut off (<0.001). This created a final sample of 334 on which the remaining checks and main analyses were undertaken. Bartlett’s test for homogeneity of variances (56248; p < 0.001), indicates that there is significant heterogeneity in variances across the groups, so the robust maximum likelihood method was used for the CFA. The Kaiser-Meyer-Olkin statistic (KMO = 0.9) and Bartlett’s test of sphericity (χ2 _(df=528)_=5155.23, p < .001) indicated that the items were factorable.

For the CFA sample, scale reliability was demonstrated through Cronbach’s alpha (α= 0.69–0.89) and McDonald’s omega (ω=0.73–0.92), suggesting at least acceptable reliability, with some subscales (Trusting and Attention Regulation) displaying excellent internal reliability [61], shown in Table 5.

The findings indicate that the 5-factor model demonstrated a good fit for the data, showing better fit than the original 8-factor model, which demonstrates a close to good fit, as displayed in Table 6.

**Table 6:** Comparison of goodness of fit measures between the original 8-factor MAIA and the 5-factor MAIA-Preg in the CFA.

	Original 8 factor model	5 factor model
SRMR	0.069	0.05
TLI	0.874	0.967
CI (RMSEA)	0.04-0.05	0.026-0.047
RMSEA	0.05	0.037
CFI	0.89	0.972
Cumulative variance	0.53	0.54
Number of questions	32	19
Chi Square (df)	948.42 (436)	207.025 (142)

The Average Variance Extracted (AVE; Table 7) demonstrates that Trust and Self-Regulation capture a large amount of the variance of both the original and the five-factor model, with Not Distracting capturing the lowest proportion of the scales variance.

**Table 7:** Average Variance Explained (AVE) for the original eight factor MAIA and the five factor MAIA-Preg.

	Original 8 factor model	5 factor model
Attention regulation	0.48	0.49
Trust	0.72	0.72
Self-Regulation	0.55	0.59
Not Distracting	0.38	0.39
Emotional Awareness	0.49	0.36
Noticing	0.31	N/A
Not worrying	0.41	N/A
Listening	0.56	N/A

### Phase 2b: Measurement invariance

Tests of measurement invariance were attempted on all 3 trimesters. There was an insufficient sample size for measurement invariance to be considered for participants in Trimester one (N = 28), therefore configural, metric and scalar tests were run just for participants in Trimester two (N = 135) and three (N = 161). Together, these results indicate that individuals in trimester 2 and trimester 3 interpret the items in the same way, and the constructs being measured remain stable across these groups. This supports the validity of the scale for assessing constructs across the later stages of gestation (see Table 8). For results separated into each Trimester see S3.

**Table 8:** Measurement invariance between trimester two and three.

	Chi Square	CFI	TLI	RMSEA (95%CI)	SRMR
Configural	Chi Square (284) = 412.07, p < .001	0.949	0.939	0.055 (0.043-0.067)	0.060
Metric	Chi Square (342) =431.983, p < .001	0.947	0.939	0.055 (0.043-0.066)	0.068
Scalar	Chi Square (312) = 443.702, p < .001	0.948	0.943	0.053 (0.042-0.064)	0.069

### Phase 3: Validation in other samples

The post-natal sample initially included 174 post-natal women, however 4 participants were identified by the Mahalanobis test as outliers, leaving a remaining sample of 170. The initial sample of 396 non-pregnant women was reduced to 387 after 9 participants were removed due to being outliers according to the Mahalanobis test (<0.001). The remaining checks and main analyses were undertaken on the remaining 387 non-pregnant participants. Tests of appropriateness for CFA suggested the datasets are large enough to correlate sufficiently and therefore are factorable (Bartlett’s test: 57.898 and 135.67, p = 0.002 and p < 0.001; KMO: 0.86 and 0.88 and Bartlett’s tests of sphericity: χ2 _(df=496)_=2935.391, p < .001 and χ2 _(df=496)_=6127, p < .001 for non-pregnant and post-natal respectively). Confirmatory factor analysis was therefore performed on the sample of post-natal and non-pregnant women, which demonstrated that the five factor model fits well, particularly for non-pregnant women, shown in Table 9.

**Table 9:** CFA outcomes for post-natal and non-pregnant samples using the five factor MAIA-MA.

	5 factor model for post-natal women	5 factor model for non-pregnant women
SRMR	0.071	0.059
TLI	0.898	0.915
CI (RMSEA)	0.057-0.083	0.056-0.072
RMSEA	0.070	0.064
CFI	0.915	0.930
Chi Square (df)	263.622	366.085

For the post-natal and non-pregnant samples, scale reliability was demonstrated through Cronbach’s alpha (α=.69-.91 and.69-.89 respectively) and McDonald’s omega (ω=.75-.91 and.69-.93 respectively), suggesting at least acceptable to excellent reliability for both comparison groups, with the scale demonstrating slightly higher internal reliability of subscales for the non-pregnant women than the post-natal women [61].

## Discussion

An EFA to CFA approach was used to explore the best-fitting model of the Multidimensional Assessment for Interoceptive Awareness (MAIA) in pregnant women. As anticipated, the original MAIA factor structure fell below good fit thresholds for our pregnant sample. The fit indices from the exploratory factor analysis, and the comparison with the original MAIA scale in the confirmatory factor analysis indicate that a five factor, 19-item model of interoceptive awareness is more appropriate for use with pregnant samples, giving rise to the MAIA-Preg. Subscales of Trusting, Attention Regulation, Self-Regulation and Not Distracting from the original MAIA remained. The one remaining question from the Noticing subscale, which related to how tension feels in the body loaded heavily with the remaining questions from the Emotional Awareness subscale, so the factor title of Emotional Awareness was retained. The Not Worrying and Body Listening scales did not remain. The 5-factor, 19-item model can therefore be seen as more appropriate to measure the interoceptive experience in pregnant participants. The new MAIA-Preg has five rather than the eight dimensions of the original MAIA, which may reflect that interoception manifests as a slightly different construct during pregnancy. These findings support other research which suggests that some constructs are experienced differently during pregnancy, for example body satisfaction [7], and pain [14], and supports that scales validated for the general population may not apply during pregnancy [42,62].

Measurement invariance across trimesters and exploration of MAIA-Preg fit for non-pregnant and post-natal samples indicates that the MAIA-Preg is a good fit, particularly for non-pregnant women, and therefore could be a useful tool to compare self-reported levels of interoceptive sensibility across different pregnant groups, or for use in longitudinal studies as it provides a valid comparison across the whole perinatal period. This can overcome the issue faced by scales designed for use in the general population not being appropriate for pregnancy and also scales designed specifically for pregnancy not being appropriate for non-pregnant groups. Having the MAIA-Preg that is valid across multiple groups means that direct comparisons can be made in terms of changes and differences in interoception. However, it is important to consider that the sample size for the postnatal group is lower than is conventionally recommended for conducting a CFA as well as the fit metrics being lower for this group (even though data checks suggested the sample was adequate for CFA). Therefore, the appropriateness of this scale for postnatal women should be addressed with caution, and requires further exploration as the interoceptive experience may be qualitatively different again during the postnatal period.

The construct of trust in the body as a safe place and trust in bodily sensations has been found to be important during pregnancy. Previous research suggests that some women report trusting their body more during pregnancy [7], particularly during their first pregnancy [29], and a recent study also found that trust increased in a pregnant sample experiencing mindfulness training but not in a control sample who did not experience the training [26]. This further indicates that trust is a fundamental element of the pregnancy interoceptive experience. Evidence also suggests that the Trust scale of the MAIA is particularly strong in a pregnant sample [30], which is reflected in the current study, showing excellent internal reliability (ω = .89) and captures the second largest proportion of the variance in the model (23%) despite only being a three item subscale. Because pregnancy is a time of substantial physical changes, many of which are beyond the control of the individual, trusting the body to ensure safe growth of the fetus [63] and during labour [64] may be particularly important. Trust has been found to be strongly related to body satisfaction during pregnancy [7,31] and postnatal outcomes [31], as well as to mediate the impact of parity (whether a woman is experiencing her first or subsequent pregnancy), and parenting status on body satisfaction [29]. The importance of trusting the body during pregnancy illustrates why this subscale would be particularly strong and accounts for a large amount of the variability when applying the MAIA to pregnant women, and further validates previous findings that use this measure in pregnancy.

Likewise the items relating to Attention Regulation and Self-Regulation subscales remained particularly strong in pregnant women. These subscales measure the ability to sustain and control attention to bodily sensations, and regulate distress through these means. During pregnancy, the ability to attend to changes in body sensations is important [65]. For example, to ensure that the pregnant person is aware of any changes to fetal movements [66] and their own body functions, such as sudden shoulder pain being a sign of potential ectopic pregnancy [NHS, 2022], and itching skin being a symptom of Cholestasis (liver disorder; [61]). Awareness of bodily sensations can be associated with reduced anxiety in pregnant women [28], which can explain why both the Attention Regulation and the Self-Regulation subscales remain strong, as they relate to the ability to regulate distress. However one question, ‘I am able to consciously focus on my body as a whole’ in the Attention Regulation subscale, did not load onto any factor, which could be because pregnant women are more likely to attend to certain sensations in specific parts of their body for example their abdomen and pelvis, rather than their body as a whole.

The items in the Not Distracting subscale also seemed to capture the construct of not distracting from body sensations during pregnancy, possibly because the questions within this subscale focus on reactions to discomfort, specifically ‘powering through’, or ignoring discomfort. Women may expect some levels of discomfort during pregnancy because of the additional weight, hormone changes and pressure on various visceral systems, so may attempt to ignore some negative sensations. However, analysis of a general population sample indicated that the Not Distracting subscale was less robust, due to having low internal consistency (α=0.69; ω = 0.73) compared to other subscales [67]. This lower internal consistency could indicate heterogeneity in experiences of deliberately attending to or avoiding signals amongst a pregnant population, which could be related to anticipation of pregnancy or past experiences of pregnancy being positive or negative.

Removing and merging the subscales related to sensing and emotions during pregnancy indicate that this is a complex and qualitatively different relationship during pregnancy, perhaps a weaker link between emotion and interoception compared with the general population. Lack of loading on emotionally based questions (e.g., ‘When I feel physical pain, I become upset’ and ‘I start to worry that something is wrong if I feel any discomfort’ in the Not Worrying subscale) might indicate that for pregnant women their bodily sensations are merely a practical, informative, element of pregnancy and they develop resilience to feeling emotions in relation to them [26,29]. The questions about listening to the body in the MAIA (e.g., ‘I listen for information from my body about my emotional state’ and ‘When I am upset, I take time to explore how my body feels’) are focussed on the emotional reactions to recognising sensations rather than the objective recognition of them, which may not accurately reflect why women listen to their body in pregnancy. Alternatively, the link between interoception and emotions might be weakened during pregnancy. For example, emotions like excitement and fear are associated with stronger and faster heartbeats. However, hormonally driven changes in the cardiac system during pregnancy mean that stronger and faster heartbeats are present in the absence of such emotions [68], thus body sensations may be less informative for emotional state at this time. Additionally, it may be that the type of worry implied in the MAIA questions is too generic as they are intended for a general population, so do not access the body worries that pregnant samples have, which may be more specifically related to the fetus and their health in relation to developing the fetus. In the original eight-factor MAIA scale, Not Worrying showed weak internal consistency in research studies of non-pregnant [67] and pregnant samples [30], so it is unsurprising that this scale dissolved.

Previous research has indicated weak internal consistency for the Noticing, Not Distracting and Not Worrying subscales of the MAIA in the general population [67], and in Noticing and Not Worrying in a pregnant sample [30], which may indicate a general instability of these subscales and is therefore unsurprising that they were excluded when statistically analysing the stability of the subscales in a different population. In the current pregnant sample, whereby subscales of Not Worrying and Noticing, as well as Body Listening were removed in the MAIA-Preg as they did not load sufficiently. A potential reason for the construct of worry in the Not Worrying subscale not translating well to pregnant samples is that the type of discomfort captured by the MAIA may not lead to additional worry in a pregnant population compared to a general population. The questions in the MAIA focus specifically on pain and discomfort signals, whereas in pregnancy many women expect some level of discomfort and pain as an anticipated and intrinsic part of the experience [69], and is therefore often attributed as being benign rather than threatening or worrying. Therefore, pregnant women may listen to body signals for the purposes of understanding the body and the fetus rather than emotional interpretations of the signals. Research also suggests that women become more skilled over the course of gestation at listening to visceral signals [70], not distracting from visceral signals [29], noticing body signals and having more body awareness [27]. Taken together, this suggests that women are less likely to consciously avoid feelings of pain and discomfort when they are pregnant, particularly in the latter weeks of pregnancy. This could be due to being more aware of the fetus, wanting to be aware of early signs of labour, the body and mind being consumed by the vast bodily changes and new sensations or just being allowed to feel the often expected discomfort caused by pregnancy. However, some literature indicates that pain tolerance reduces in pregnancy, particularly in those who fear labour [71] and that pain correlates with emotional state more in pregnant women than non-pregnant women [14]. In addition to direct responses to physical sensations, research indicates that one element of interoception as measured by the MAIA subscale, ‘trust in the body’, changes more than others during the course of pregnancy [7]. This suggests that measures of internal sensations that are not validated for use in pregnancy may not recognise the specific experiences of pain and interpretation of pain at this time. Therefore, in addition to the current MAIA-Preg that is suitable across pregnant, post-natal and non-pregnant groups additional measures of well defined constructs are needed that are specifically designed for pregnancy.

This suggests that several subscales could benefit from further exploration in pregnant samples to discern if they in fact are clearly different constructs entirely, or if the noted differences are due to slight changes in nuance of the question. Future research might consider clearer definitions of sensations to differentiate those that cause concern or worry and those that are expected and/or accepted, as well as scales specifically designed for pregnant women would help to understand those nuances of the interoceptive experience during pregnancy.

### Limitations of the methodology

Although the sample was relatively large, as is often the case in perinatal research, white, middle class and educated women dominated the sample despite proactive efforts to recruit minority voices. Hearing minority voices needs to be continually strived for in pregnancy research as the pregnancy experience [72] and the interoceptive experience [73] can differ vastly between different ethnic and societal groups. Likewise, there were limited numbers of participants in Trimester one, which was demonstrated in measurement invariance testing, which could be due to women in early pregnancy not knowing they are pregnant or not wanting to engage in research due to insecurity about the viability of their early pregnancy. The lack of women responding to the scale in the first trimester could reduce the validity of the scale across gestation, given that physiological and interoceptive experience are likely different across trimesters.

## Conclusion

This study explored the factor structure of the MAIA in pregnant women using exploratory and confirmatory factor analysis. The adjustment to the factor structure of the original MAIA indicates key differences in the interoceptive experience during pregnancy compared with the general population that are absent from the original MAIA scale. A five-factor 19-item model was supported, and demonstrated better indices of fit than the original eight-factor, 32-item model. Overall, the MAIA-Preg may be a valid measure for understanding interoceptive sensibility during at least trimester two and three of pregnancy, and to compare across the perinatal period [31] and non-pregnant samples. Due to the importance of interoception in mental well-being, the scale has the implications for use both for research purposes as well as in antenatal medical settings for better understanding bodily experiences during pregnancy on an individual basis. This understanding could inform more tailored and effective interventions and personalised antenatal care plans, for example by identifying people early in pregnancy who have poor interoceptive experiences and therefore may be at heightened risk of mental ill-health during pregnancy. Such screening may help inform interventions designed to improve or enhance attention to internal signals, such as mindfulness [74] or yoga [75].

By accurately assessing interoceptive sensibility in early-mid pregnancy, mental health outcomes could be improved by potentially identifying and mitigating early signs of perinatal mental illness, or via the implementation of education programs for expectant mothers to help them understand and manage their bodily changes during pregnancy.

## Supporting information

S1 File The MAIA-Preg five factor model for pregnant women. (DOCX)

S2 File Factor loadings for MAIA questions. (DOCX)

S3 File CFI and TLI for the total sample and for participants from trimester two and three. (DOCX)
