# Supplementary material for: Analysing the factor structure of the MAIA scale for pregnant women: Development of the MAIA-Preg
Source: PLoS One. 2025 May 7;20(5):e0322499. doi: 10.1371/journal.pone.0322499 (PMC12058024; doi:10.1371/journal.pone.0322499)
Supplement: S1 File — (DOCX) [file pone.0322499.s001.docx]

**Supporting information**

**S1: The MAIA-Preg five factor model for pregnant women.**

| **Subscale** | **Question number** | **Question** |
| --- | --- | --- |
| **Not distracting** | 1* | I do not notice (I ignore) physical tension or discomfort until they become more severe. |
|  | 2* | I distract myself from sensations of discomfort. |
|  | 3* | When I feel pain or discomfort, I try to power through it. |
| **Attention regulation** | 4 | I can pay attention to my breath without being distracted by things happening around me. |
|  | 5 | I can maintain awareness of my inner bodily sensations even when there is a lot going on around me. |
|  | 6 | When I am in conversation with someone, I can pay attention to my posture. |
|  | 7 | I can return awareness to my body if I am distracted. |
|  | 8 | I can refocus my attention from thinking to sensing my body |
|  | 9 | I can maintain awareness of my whole body even when a part of me is in pain or discomfort. |
| **Emotional Awareness** | 10 | When I am tense I notice where the tension is located in my body |
|  | 11 | I notice how my body changes when I am angry. |
|  | 12 | When something is wrong in my life I can feel it in my body |
|  | 13 | I notice how my body changes when I feel happy / joyful. |
| **Self-regulation** | 14 | When I bring awareness to my body I feel a sense of calm |
|  | 15 | I can use my breath to reduce tension. |
|  | 16 | When I am caught up in thoughts, I can calm my mind by focusing on my body/breathing. |
| **Trusting** | 17 | I am at home in my body. |
|  | 18 | I feel my body is a safe place. |
|  | 19 | I trust my body sensations. |
