# Supplementary material for: Analysing the factor structure of the MAIA scale for pregnant women: Development of the MAIA-Preg
Source: PLoS One. 2025 May 7;20(5):e0322499. doi: 10.1371/journal.pone.0322499 (PMC12058024; doi:10.1371/journal.pone.0322499)
Supplement: S2 File — (DOCX) [file pone.0322499.s002.docx]

**Supporting information**

**S2: Factor loadings for MAIA questions**

| **MAIA question number** | **MAIA subscale** | **MAIA question (MAIA-Preg question number, and reverse scoring*)** | **Loading 1** | **Loading 2** | **Loading 3** | **Loading 4** | **Loading 5** | **MAIA-Preg subscale** |
| --- | --- | --- | --- | --- | --- | --- | --- | --- |
| 1 | Noticing | When I am tense I notice where the tension is located in my body. (10) | -0.08 | -0.01 | 0.17 | 0 | **0.33** | **Awareness** |
| 2 |  | I notice when I am uncomfortable in my body. |  |  |  |  |  |  |
| 3 |  | I notice where in my body I am comfortable. |  |  |  |  |  |  |
| 4 |  | I notice changes in my breathing, such as whether it slows down or speeds up. |  |  |  |  |  |  |
| 5 | Not distracting | I do not notice (I ignore) physical tension or discomfort until they become more severe. (1 *) | **0.44** | 0.07 | -0.12 | 0.14 | 0 | **Not distracting** |
| 6 |  | I distract myself from sensations of discomfort. (2 *) | **1** | -0.01 | 0.02 | -0.02 | 0 | **Not distracting** |
| 7 |  | When I feel pain or discomfort, I try to power through it. (3 *) | **0.4** | -0.03 | 0.09 | 0.08 | -0.04 | **Not distracting** |
| 8 | Not worrying | When I feel physical pain, I become upset. |  |  |  |  |  |  |
| 9 |  | I start to worry that something is wrong if I feel any discomfort. |  |  |  |  |  |  |
| 10 |  | I can notice an unpleasant body sensation without worrying about it. |  |  |  |  |  |  |
| 11 | Attention regulation | I can pay attention to my breath without being distracted by things happening around me. (4) | 0.04 | 0.21 | -0.01 | **0.51** | -0.16 | **Attention regulation** |
| 12 |  | I can maintain awareness of my inner bodily sensations even when there is a lot going on around me. (5) | 0.03 | -0.11 | -0.01 | **0.75** | 0.07 | **Attention regulation** |
| 13 |  | When I am in conversation with someone, I can pay attention to my posture. (6) | -0.02 | -0.07 | 0.04 | **0.66** | 0.03 | **Attention regulation** |
| 14 |  | I can return awareness to my body if I am distracted. (7) | 0.05 | 0.08 | 0.02 | **0.72** | 0.08 | **Attention regulation** |
| 15 |  | I can refocus my attention from thinking to sensing my body. (8) | -0.04 | 0.09 | -0.02 | **0.76** | -0.07 | **Attention regulation** |
| 16 |  | I can maintain awareness of my whole body even when a part of me is in pain or discomfort. (9) | -0.05 | 0.02 | 0.18 | **0.56** | 0.07 | **Attention regulation** |
| 17 |  | I am able to consciously focus on my body as a whole. |  |  |  |  |  |  |
| 18 | Emotional awareness | I notice how my body changes when I am angry. (11) | 0.07 | 0.07 | -0.03 | 0 | **0.6** | **Awareness** |
| 19 |  | When something is wrong in my life I can feel it in my body. (12) | -0.06 | -0.02 | 0.01 | 0.03 | **0.7** | **Awareness** |
| 20 |  | I notice that my body feels different after a peaceful experience. |  |  |  |  |  |  |
| 21 |  | I notice that my breathing becomes free and easy when I feel comfortable. |  |  |  |  |  |  |
| 22 |  | I notice how my body changes when I feel happy / joyful. (13) | 0.06 | 0.22 | -0.05 | 0.17 | **0.39** | **Awareness** |
| 23 | Self-regulation | When I feel overwhelmed I can find a calm place inside. |  |  |  |  |  |  |
| 24 |  | When I bring awareness to my body I feel a sense of calm. (14) | -0.04 | **0.48** | 0.14 | 0.09 | 0.19 | **Self-regulation** |
| 25 |  | I can use my breath to reduce tension. (15) | 0.01 | **0.94** | 0.01 | -0.04 | -0.01 | **Self-regulation** |
| 26 |  | When I am caught up in thoughts, I can calm my mind by focusing on my body/breathing. (16) | 0.02 | **0.69** | 0.04 | 0.15 | 0.06 | **Self-regulation** |
| 27 | Body listening | I listen for information from my body about my emotional state |  |  |  |  |  |  |
| 28 |  | When I am upset, I take time to explore how my body feels. |  |  |  |  |  |  |
| 29 |  | I listen to my body to inform me about what to do |  |  |  |  |  |  |
| 30 | Trusting | I am at home in my body. (17) | 0.02 | -0.03 | **0.84** | 0.08 | -0.06 | **Trust** |
| 31 |  | I feel my body is a safe place. (18) | 0 | -0.01 | **0.95** | -0.02 | -0.02 | **Trust** |
| 32 |  | trust my body sensations. (19) | 0.02 | 0.15 | **0.7** | -0.03 | 0.14 | **Trust** |
