# Supplementary material for: Analysing the factor structure of the MAIA scale for pregnant women: Development of the MAIA-Preg
Source: PLoS One. 2025 May 7;20(5):e0322499. doi: 10.1371/journal.pone.0322499 (PMC12058024; doi:10.1371/journal.pone.0322499)
Supplement: S3 File — (DOCX) [file pone.0322499.s003.docx]

**Supporting information**

**S3: CFI and TLI for the total sample and for participants from trimester two and three**

| Trimester (N) | CFI | TLI | RMSEA |
| --- | --- | --- | --- |
| 1 (28) | N/A (inadequate sample size) | N/A (inadequate sample size) | N/A (inadequate sample size) |
| 2 (135) | .951 | .942 | 0.055 |
| 3 (161) | .947 | .936 | 0.055 |
| Whole model | .95 | .939 | 0.04 |
